# Toward a Positive Life beyond COVID-19: Problem-Solving Appraisal as a Resistance Resource in the Relationship between Stress and Well-Being in Students

**DOI:** 10.3390/healthcare11030350

**Published:** 2023-01-25

**Authors:** Tyrone B. Pretorius, Anita Padmanabhanunni

**Affiliations:** Department of Psychology, University of the Western Cape, Bellville 7530, South Africa

**Keywords:** problem-solving appraisal, perceived stress, health-sustaining, stress-buffering, hopelessness, anxiety

## Abstract

The COVID-19 pandemic is a major global stressor that has been associated with elevated risk of negative mental health symptoms. As a helping profession, our main task should be identifying factors that may shield individuals from the negative consequences of stress, rather than only focusing on the causes and symptoms of stress. One such factor, identified in the literature, is an individual’s perception of their problem-solving skills. In this study we investigate the role of problem-solving appraisal in the association between perceived stress and psychological well-being. Participants were students (*n* = 322) who completed the Problem Solving Inventory, the Perceived Stress Scale, the short forms of the trait scale of the State-Trait Anxiety Inventory, and the Beck Hopelessness Scale. The results demonstrate the health-sustaining benefits of problem-solving appraisal, as all dimensions of problem-solving appraisal (problem-solving confidence, approach-avoidance style, and problem-solving control) were directly associated with hopelessness and anxiety. The stress-buffering role of problem-solving appraisal with respect to hopelessness was demonstrated through the interaction between perceived stress and problem-solving appraisal. However, problem-solving appraisal did not buffer the effects of stress on anxiety. The findings indicate that problem-solving appraisal may be an important protective resource that could be beneficial for coping with other negative events and experiences beyond COVID-19. The implications of these findings for interventions are discussed.

## 1. Introduction

The COVID-19 outbreak is a major stressor that has significantly impacted global mental health. Longitudinal studies (e.g., [1,2]) have reported an overall increase in mental health disorder prevalence from pre-COVID-19 to during the pandemic. Elevated levels of mental health-related symptoms have been found to be most pronounced among young university-aged adults [1]. Disruptions to the higher education system and students’ academic experiences have been a salient feature of the pandemic and such disruptions are thought to underlie the heightened levels of stress encountered among this population group [3,4]. At the onset of the pandemic, students had to negotiate the abrupt closure of campuses and the rapid transition to emergency remote learning. Financial difficulties and limited access to digital resources as well as disruptions to social networks and peer relationships added to the stressors experienced by students during this time [3,4,5]. Further, students’ concerns about the impact of the pandemic on their academic progress, fears of contagion, and fears of transmitting the virus to loved ones have proven to heighten levels of distress [4].

The current study was undertaken at a historically disadvantaged South African institution. The term historically disadvantaged institution (HDI) is used to describe universities that were created under the apartheid system of governance for black South Africans. These universities were severely under-resourced and despite significant post-apartheid shifts, the student population at these institutions continues to be from predominantly lower working-class groups [6]. Students at HDIs are more likely to reside in low-income communities characterized by high levels of unemployment, poverty, substance abuse and gang violence [6,7]. It has been well-established that the COVID-19 pandemic differentially impacted low-to-middle-income countries as well as under-resourced communities within high-income settings [7,8]. This is due to the significant discrepancies in access to basic resources, including health facilities, clean running water and personal protective equipment [7]. South African studies undertaken in 2020–2022 [9] have provided evidence of the differential impact of the pandemic on students residing in disadvantaged community contexts and highlighted elevated levels of mental health disorders. Although COVID-19-related containment measures were largely rescinded in 2022, these measures and related policy decisions had unprecedented effects on economic, educational and social sectors in the country and contributed to heightened levels of strain [7]. According to the stressor-strain theory [10], chronic exposure to stress can lead to the development of mood and anxiety disorders. Prior to the pandemic, students who reported higher stress than peers were also found to report higher levels of depression, anxiety, and somatization [11]. In addition, stress among students was associated with common mental disorders such as major depressive disorder, bipolar disorder, generalized anxiety disorder, and panic disorder, among others [12]. Similarly, during the COVID-19 pandemic, stress has been associated with anxiety [13,14], physical and psychological health as measured by the Chinese Health Questionnaire [3], and depressive symptoms [14].

In sum, the relationship between stress and psychological well-being is well documented. Thus, in our view, little is to be gained by continuing research on this association in different populations. As members of a helping profession, it is our responsibility to minimize the detrimental effects of stress on well-being. The existing literature predominantly focuses on stress management approaches, which are interventions that focus on either minimizing the sources of stress or the impact of stress. Holman and colleagues [15] provide a useful typology of stress management interventions within organizational settings, describing interventions as either focused on the individual or at an organizational level. Individual and organizational interventions may be further classified as primary, secondary, or tertiary. Primary interventions are largely preventive and focus on the sources of stress, secondary interventions attempt to reduce the severity and duration of stress, and tertiary interventions attempt to address the consequences of stress for individuals who have already suffered detrimental impacts. Holman and colleagues [15] summarize the available meta-analytic studies and qualitative reviews of stress management interventions and conclude that there is convincing evidence to support the efficacy of these interventions.

The same typology may be applied to the stress experienced by students at a university or college. In this regard, buddy systems [16] and peer mentoring [17], implemented in relation to stress, can be regarded as exemplars of secondary organizational-level interventions. Kachaturoff and colleagues [17] conducted an integrative review of the effect of peer mentoring on the stress experienced by undergraduate students and concluded that peer mentoring decreased students’ stress. Conversely, interventions aimed at reducing the consequences of stress (e.g., cognitive behavioral therapy) may be regarded as secondary individual-level interventions. For example, Lemay and colleagues [18] examined the efficacy of yoga and meditation to reduce the stress and anxiety experienced by pharmacy students and found that these interventions were successful at reducing anxiety.

Another line of research has focused on other variables that influence or impact the relationship between stress and psychological well-being. Examples of such variables include social support [19], ego resilience [20], resilience [21], hardiness [22], and sense of coherence [23]. These variables may make individuals differentially vulnerable to adverse events. This line of research is useful because it identifies variables that can serve as protective factors in other adverse situations, not only in the face of stress. For example, Shumaker and Brownell [24] propose a conceptual model of social support that distinguishes between the health-sustaining and stress-buffering roles of social support. With regards to the health-sustaining role they suggest that support is directly related to well-being and expressed the view that support is important for well-being both in the presence and absence of stress. The stress-buffering is also referred to as the moderating role. In the stress-buffering model, the third variable (e.g., social support) influences the strength and direction of the relationship between stress and well-being, such that the relationship between stress and well-being is strong at low levels of social support and weak at high levels.

The focus of the current study is on problem-solving appraisal, a variable that is presumed to have a health-sustaining and stress-buffering function. Problem-solving is defined as the self-directed cognitive-behavioral process through which individuals attempt to identify effective solutions for specific problems [25]. These problems or stressors can be everyday life events or major negative experiences (e.g., exposure to trauma, job loss, or the death of a loved one) that require substantive readjustments in a person’s life [25]. Emotional responses to stressors are significantly influenced by cognitive appraisals of the event (e.g., as threatening or as a challenge to be overcome) and appraisals of the individual’s ability to cope effectively [26]. Problem-solving appraisal refers to individuals’ perception of the effectiveness of their problem-solving skills [27]. It consists of three dimensions, namely problem-solving confidence (PSC; individuals’ trust in and self-assurance about their problem-solving abilities), approach-avoidance style (AAS; the tendency to approach or avoid problem-solving activities), and personal control (PC; the belief that one is in control of one’s emotions and behaviors while solving problems) [27]. In 2004, Heppner and colleagues [28] conducted a comprehensive review and synthesis of the relationship between problem-solving appraisal and psychological adjustment. They concluded that evidence supports a strong association between problem-solving appraisal and a wide range of indices of psychological adjustment. More recent studies, (e.g., [29,30,31]) have reported moderate to strong associations between problem-solving appraisal and indices of psychological well-being in different samples. Abdollahi and colleagues [29] reported a strong association between problem-solving appraisal and suicide ideation (*r* = 0.69) in a sample of nursing students in Malaysia, while Teo and colleagues [30] found a moderate relationship between problem-solving appraisal and suicide ideation (*r* = 0.34) in a sample of young adult males in Singapore. They also reported a moderate association between problem-solving appraisal and depression (*r* = 0.40). Pretorius [31] found a strong correlation between problem-solving appraisal and anxiety (*r* = 0.52) in a sample of secondary school teachers.

The current study is grounded in the theoretical model of stress-buffering which proposes that protective factors can mitigate the associations between adverse or stressful live events and mental health outcomes [32]. The aim of the current study is to examine the health-sustaining and stress-buffering roles of the dimensions of problem-solving appraisal in relation to hopelessness and anxiety. Regarding the health-sustaining role of problem-solving appraisal, we hypothesized that the direct effects of PSC, AAS, and PC on hopelessness and anxiety would be significant. Regarding the stress-buffering role of problem-solving appraisal, we hypothesized that PSC, AAS, and PC would significantly interact with perceived stress to impact hopelessness and anxiety.

## 2. Materials and Methods

### 2.1. Participants and Procedure

Participants were students (*n* = 322) at a metropolitan university in the Western Cape province of South Africa. Data were collected in 2022. The University continued a blended learning and teaching approach in 2022 to promote COVID-19-related safety protocols. We used Google Forms to develop a web-based version of the instruments used in the current study. Participants were randomly selected by the office of the Registrar of the university. The details of all students enrolled in the university were captured in an Excel spreadsheet. An algorithm was then used to randomly select 1700 students. An email with a request to participate in the study and a link to the survey was sent to these students. The participants were predominantly women (77%) and resided in an urban area (87.3%). The mean age of the sample was 26.01 years (SD = 10.19). In all, 25.5% of the sample had previously tested positive for COVID-19, and 40.7% had lost a family member to COVID-19.

### 2.2. Instruments

Participants completed a brief demographic questionnaire, as well as the following instruments: the Perceived Stress Scale (PSS) [33], the Problem Solving Inventory (PSI) [27], a short form of the Beck Hopelessness Inventory (BHS-9) [34], and the 5-item version of the trait scale of the State-Trait Anxiety Inventory (STAI-T5) [35]. The PSS is a 10-item measure of the extent to which individuals perceive their lives to be stressful and is scored on a 5-point scale ranging from 0 (Never) to 4 (Very Often). An example item of the PSS is: “*In the last month, how often have you been able to control irritations in your life?*” The PSS development study reported reliability estimates (Cronbach’s alpha) ranging from 0.84 to 0.86. In addition, the validity of the PSS has been demonstrated through correlations between perceived stress and life-event scores, as well as depressive and physical symptomatology [33]. In South Africa, Steyn and Vawda [36] reported a reliability coefficient of 0.87 for the PSS when used with a sample of workers in large organizations.

The PSI is a 32-item measure of participants’ perceptions of their own problem-solving skills and is scored on a 6-point Likert scale ranging from 1 (Strongly Agree) to 6 (Strongly Disagree). The PSI is scored such that high scores are indicative of perceptions of ineffective problem-solving skills. The scale consists of three dimensions: problem-solving confidence (PSC), approach-avoidance style (AAS), and personal control (PC). Example items of the three dimensions are: “*When I make plans to solve a problem, I am almost certain I can make them work*” (PSC), “*I generally go with the first idea that comes to mind*” (AAS), and “*I make snap judgements and later regret them*” (PC). Heppner and Petersen [27] reported satisfactory reliability coefficients for the three dimensions, ranging from 0.72 to 0.85. In addition, the relationship between problem-solving appraisal and participants’ ratings of their problem-solving skills served as evidence of validity. In South Africa, similarly satisfactory reliability coefficients were reported (PSC: *α* = 0.79, AAS: *α* = 0.84, PC: *α* = 0.71) [37]. In addition, problem-solving appraisal was found to be related to family environment [38] and interacted with social support to buffer the effect of stress on depression [39].

The BHS-9 is a 9-item short version of the original 20-item Beck Hopelessness Scale (BHS) [40], which was developed using item response theory. The 9 items are scored on a dichotomous Yes/No scale. An example item of the BHS-9 is: “*Things just don’t work out the way I want them to*.” In the original study that reported on the development of the 9-item version, Balsamo and colleagues [34] reported satisfactory reliability coefficients (Mokken scale reliability = 0.86, *α* = 0.86, latent class reliability coefficient = 0.89). A Mokken analysis also confirmed that the BHS-9 is a unidimensional scale. The full version of the BHS has been used in studies in South Africa, and reliability coefficients of 0.89 have been reported for a student sample [41] and a teacher [42] sample.

The STAI-T5 is a 5-item version of the original 20-item trait scale of the State-Trait Anxiety Inventory (STAI-T) [43]. It is scored on a 4-point scale ranging from 1 (Not at all) to 4 (Very much so). An example item of the STAI-T5 is: “*I take disappointments so keenly that I can’t put them out of my mind*.” The authors of the short version of the STAI reported a reliability coefficient of 0.82 and found that the psychometric properties of the short form were generally comparable to those used for the 20-item version. Correlations between the short version of the STAI-T and depression, life satisfaction, and self-esteem serve as evidence for its validity [35]. In South Africa, satisfactory reliability coefficients have been reported for the 20-item version of the STAI-T for a student sample (*α* = 0.90) [9] and a teacher sample (*α* and *ω* = 0.91) [9].

### 2.3. Ethics

The study was conducted in accordance with the guidelines of the Declaration of Helsinki and was approved by the Humanities and Social Sciences Ethics Committee of the University of the Western Cape (ethics reference number: HS22/2/9, February 2022). Participants provided informed consent on the landing page of the electronic link. Participation was voluntary and anonymous.

### 2.4. Data Analysis

Descriptive statistics (means and SD), reliabilities (alpha and omega), and the intercorrelation between study variables (Pearson’s *r*) were obtained using IBM SPSS for Windows version 28 (IBM Corp., Armonk, NY, USA). The OMEGA macro for SPSS [44] was used to determine omega. To examine the health-sustaining and stress-buffering roles of problem-solving appraisal, the PROCESS macro for SPSS [45], a regression-based approach, was used. The variables used to create the interaction term were mean-centered. The nature of the interaction effect was examined using plots generated by the visualization code supplied by PROCESS. The subgroups used in the plots were created using 1 SD below the mean (effective problem-solving), mean, and 1 SD above the mean (ineffective problem-solving).

## 3. Results

The intercorrelations, descriptive statistics and reliabilities are reported in Table 1.

The reliabilities reported in Table 1 are all at an acceptable level (*α* and *ω* > 0.70). We used Cohen’s descriptors of effect size (weak, moderate, strong) to describe the strength of associations [46]. The findings in Table 1 also reflect a significant positive association between perceived stress and hopelessness (*r* = 0.47, *p* < 0.001) and anxiety (*r* = 0.60, *p* < 0.001). In the case of hopelessness, the relationship may be considered moderate, whereas in the case of anxiety, it may be considered strong. Thus, high levels of perceived stress were associated with high levels of hopelessness and anxiety. There was also a significant positive relationship between PSC, AAS, and PC, on the one hand, and hopelessness (PSC: *r* = 0.60, *p* < 0.001; AAS: *r* = 0.33, *p* < 0.001; PC: *r* = 0.39, *p* < 0.001) and anxiety (PSC: *r* = 0.51, *p* < 0.001; AAS: *r* = 0.26, *p* < 0.001; PC: *r* = 0.54, *p* < 0.001) on the other hand. The relationships between AAS and hopelessness, PC and hopelessness, and AAS and anxiety, were moderate. In other instances, the relationships may be regarded as strong. Higher scores on the PSI are indicative of perceptions of ineffective problem-solving; thus, the observed relationships indicate that perceptions of ineffective problem-solving skills were associated with low levels of psychological well-being. Perceived stress was also positively associated with PSC (*r* = 0.54, *p* < 0.001), AAS (*r* = 0.32, *p* < 0.001), and PC (*r* = 0.51, *p* < 0.001). The relationship was moderate in the case of AAS and strong in the cases of PSC and PC. Thus, perceptions of effective problem-solving skills were associated with low perceived stress levels.

We examined whether those who tested positive for COVID-19 or those who had lost a family member due to COVID-19 differed from those who had not tested positive or lost a family member on any of the variables of interest. It is possible that these two experiences (testing positive or losing a family member) may have impacted their perception of stress related to COVID-19. The results are presented in Table 2.

The findings in Table 2 indicate that there were no significant differences between those who had tested positive and those who had not in terms of any of the variables. However, participants who had lost a family member differed significantly from those who had not in terms of stress (*t* = 3.34, *p* < 0.001) and anxiety (*t* = 2.12, *p* = 0.035). Specifically, those who had lost a family member reported higher levels of perceived stress (mean = 25.27, SD = 6.10) and anxiety (mean = 12.95, SD = 4.11) than those who had not lost a family member (stress: mean = 22.93, SD = 6.23; anxiety: mean = 11.96, SD = 4.11).

Given these observed differences, the variable “Have you lost a family member as a result of COVID-19?” was added to the moderation analysis as a covariate in the analyses pertaining to anxiety. The results of the PROCESS analyses are reported in Table 3.

The findings reported in Table 3 indicate that apart from the relationship between approach-avoidance style and anxiety, all the dimensions of problem-solving appraisal had significant direct effects on hopelessness and anxiety. More specifically, PSC had significant direct effects on hopelessness (*B* = 0.074, *p* < 0.001) and anxiety (*B* = 0.141, *p* < 0.001), AAS had a significant direct effect on hopelessness (*B* = 0.039, *p* < 0.001), and PC had a significant direct effect on hopelessness (*B* = 0.124, *p* < 0.001) and anxiety (*B* = 0.258, *p* < 0.001).

In addition, the findings in Table 3 indicate that all the interaction effects were significant in terms of hopelessness but not anxiety, which points to the stress-buffering role of problem-solving appraisal. More specifically, the interaction between perceived stress and PSC on hopelessness was significant (*B* = 0.005, *p* = 0.005), the interaction between perceived stress and AAS on hopelessness was significant (*B* = 0.006, *p* < 0.001), and the interaction between perceived stress and PC on hopelessness was significant (*B* = 0.015, *p* < 0.001).

**Figure 1:**
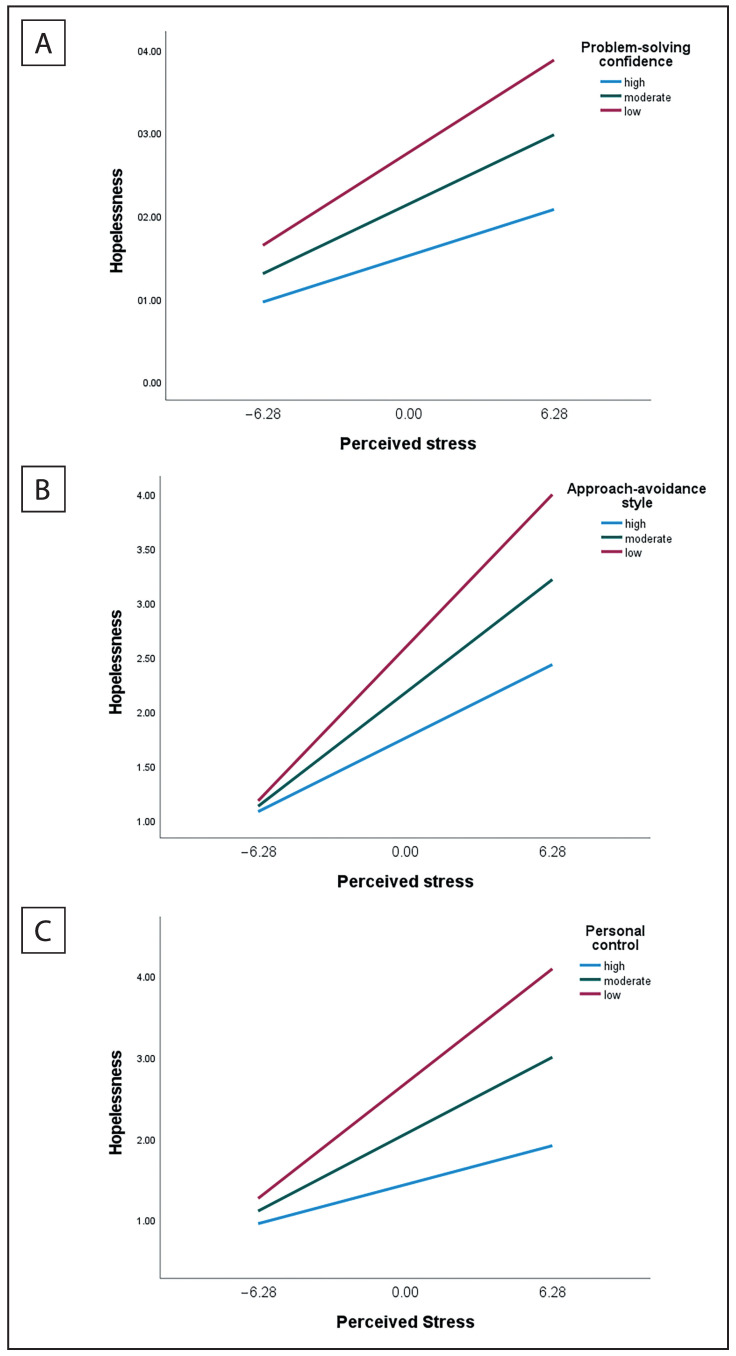
The relationship between perceived stress and hopelessness at different levels of the dimensions of problem-solving appraisals. (**A**) = problem-solving confidence, (**B**) = approach-avoidance style, (**C**) = personal control.

The nature of these interactions is illustrated in Table 4 and Figure 1.

The findings in Table 4 illustrate that while the effect was significant at all three levels of the dimensions of problem-solving appraisal, the size of the effect decreased as perceptions of problem-solving effectiveness increased. The results show that at low levels of problem-solving confidence, the effect was larger (*B* = 0.178, *p* < 0.001) than at high levels of problem-solving confidence (*B* = 0.089, *p* < 0.001). Similarly, at low levels of approach-avoidance style, the effect was larger (*B* = 0.225, *p* < 0.001) than at high levels of approach-avoidance style (*B* = 0.108, *p* < 0.001), and at low levels of personal control, the effect was larger (*B* = 0.225, *p* < 0.001) than at high levels of personal control (*B* = 0.076, *p* < 0.001).

The nature of the interaction effects is further illustrated in Figure 1.

Figure 1 illustrates that at low levels of PSC, AAS, and PC, the relationship between perceived stress and hopelessness was stronger than at high levels of PSC, AAS, and PC. At both low and high levels of perceived stress, participants with high levels of PSC, AAS, and PC reported lower levels of hopelessness.

## 4. Discussion

The COVID-19 pandemic introduced a range of unprecedented stressors, many of which have disproportionately impacted the mental health and well-being of young adults [2]. As a subgroup of the population, university students have been impacted by the COVID-19 outbreak in distinctive ways due to the rapid closure of universities and transition to emergency remote online learning, as well as social isolation from peers and limited social support [47]. The increased stress associated with the pandemic has been linked to heightened levels of anxiety, depression, substance use, hopelessness, and loneliness among this group [48]. Although exposure to multiple stressors is typically associated with a range of mental health challenges, many people do not develop psychological disorders or demonstrate functional impairments. According to the transactional model of stress, cognitive appraisals play a central role in influencing mental health outcomes [49,50]. Adaptive reappraisal of challenging situations and positive appraisals of one’s ability to problem-solve and effectively negotiate the stressor have been consistently associated with enhanced mental health outcomes. Positive appraisals act as a buffer between subjective experiences of stress and adverse outcomes, while maladaptive appraisals predict high levels of anxiety, depression, and hopelessness [49,51]. The aim of the current study was to examine the health-sustaining and stress-buffering roles of the dimensions of problem-solving appraisal in relation to hopelessness and anxiety. There were several important findings.

First, consistent with prior research, the study confirmed that high levels of perceived stress were associated with high levels of psychological distress in the form of heightened hopelessness and anxiety. Similar results have been reported in other studies conducted during the pandemic (e.g., [52]). For example, in a Turkish study, Aslan and colleagues [52] found that perceived stress among college students was significantly linked to generalized anxiety and reduced life satisfaction. Similarly, studies among US college students [53,54] have reported that high levels of perceived stress were associated with anxiety, depression, loneliness, and increased alcohol use. Sources of perceived stress during the COVID-19 pandemic identified in the literature include concerns about an economic downturn, parental job loss, limited access to personal protective equipment, fear of infection, food insecurity, and uncertainty about the future [9]. It is likely that students in the current study sample experienced similar sources of stress.

Second, the study confirmed that negative appraisals of problem-solving ability were associated with heightened hopelessness and anxiety, whereas positive appraisals of PSC, AAS, and PC were associated with reduced distress. Diminished problem-solving appraisal has been consistently identified as a predictor of adverse mental health outcomes [55,56]. For example, Dixon and colleagues [57] found that problem-solving appraisal and negative life stress were significant independent predictors of hopelessness and suicidal ideation among university students. Another study [58] reported similar results among high school students. In a meta-analytic review, Schäfer and colleagues [59] found that diminished problem-solving ability predicted anxiety and depression among youth. These findings suggest that developing problem-solving skills and promoting a positive orientation towards problem-solving may form an important part of interventions among vulnerable population groups.

Third, PSC, AAS, and PC interacted with perceived stress to impact hopelessness. This finding indicates that all three dimensions of problem-solving appraisal were found to play a stress-buffering and health-sustaining role in relation to hopelessness. According to social cognitive theory [26], individuals who appraise themselves as having effective problem-solving abilities are cognitively better able to identify and implement effective strategies to manage stressors than those who perceive themselves to be ineffective problem solvers. It remains unclear why people with a perceived effective problem-solving ability are better able to ward off anxiety and hopelessness than peers. One possible explanation is related to the broaden-and-build theory of positive emotion, which suggests that reappraising stressors as challenges that can be overcome and perceiving oneself as capable of managing adversity can lead to positive emotions, such as hope [60]. These positive emotions have the potential to widen the range of coping strategies that come to mind and consequently enhance resilience [60].

Finally, problem-solving appraisal did not buffer the effect of stress on anxiety. This can be explained by Lazarus and Folkman’s [61] goodness-of-fit hypothesis, which proposes that coping entails a good match between the characteristics of the situation and the coping strategies deployed by the individual. An appropriate level of fit leads to better outcomes in negotiating the stressor, whereas an inappropriate fit can result in heightened levels of anxiety. In the context of the COVID-19 pandemic, research [62] has demonstrated that situation-specific coping strategies impacted on anxiety levels. The use of problem-focused strategies tend to predict better adjustment in controllable situations, whereas emotion-focused strategies such as avoidance are preferable in uncontrollable situations [62]. Since the COVID-19 pandemic represents an uncontrollable situation, it is likely that individuals who prefer a problem-focused strategy may find that their efforts do not produce the desired results, and this could lead to further anxiety [63]. Furthermore, individuals with heightened levels of anxiety tend to have cognitive biases that result in ambiguous information being interpreted in a more threatening way [13]. Heightened anxiety, in turn, adversely impacts on the selection and implementation of coping responses [13]. The current study builds on the existing research on stress and coping and has important practical implications. The findings indicate that guiding young adults to positively reappraise their problem-solving ability in response to stressors and training them to use appropriate cognitive and emotional coping strategies could potentially buffer against the development of adverse mental health outcomes. Online cognitive-behavioral skills training programs with university students have proven to be effective at promoting coping and reducing anxiety and depression [64]. Further, the PSI has been effectively used as a training tool to increase awareness of problem-solving appraisals and attitudes toward problem-solving [65]; thus, this too could also be used to inform psycho-educational programs aimed to enhance student coping. Early screening of incoming students could help to identify those experiencing heightened levels of stress and aid in targeted intervention efforts to improve problem-solving appraisals, which in turn could promote adaptation and reduce distress [64].

The study has several limitations. The cross-sectional design limits the extent to which causal inferences can be drawn, and longitudinal studies are recommended to further elucidate the relationship between stressors and coping responses. Second, it is likely that participants’ appraisals and emotional responses may have varied depending on the nature of the stressors they experienced during the pandemic, and this variation may impact the generalizability of the findings. Third, the sample comprised of university students and may be subject to self-selection bias. It is possible that students with an interest in the topic may have been more likely to participate than their peers. Future studies should recruit a diverse sample to confirm the findings. Finally, it is likely that factors unrelated to the COVID-19 pandemic may have contributed to the stressors experienced by students; therefore, the results should be interpreted with caution.

## 5. Conclusions

The present study extends the literature on stress and coping by identifying a protective factor that can potentially buffer against the development of adverse mental health outcomes among university students. The study findings indicate that problem-solving appraisals can provide an effective method to regulate distressing emotions evoked by stressful life events and thereby counter against mental health problems such as anxiety and hopelessness. In this regard, problem-solving appraisal may be an effective resource, not only with respect to COVID-19, but also in relation to other negative life events and experiences. Interventions that improve problem-solving appraisals may be of significant benefit in helping students effectively negotiate the stressors associated with the pandemic.

## Figures and Tables

**Table 1:** Intercorrelations, descriptive statistics, and reliabilities.

	1	2	3	4	5	6
1. Perceived stress	–					
2. Problem-solving confidence	0.54 **	–				
3. Approach-avoidance style	0.32 **	0.60 **	–			
4. Personal control	0.51 **	0.53 **	0.50 **	–		
5. Hopelessness	0.47 **	0.46 **	0.33 **	0.39 **	–	
6. Anxiety	0.60 **	0.51 **	0.26 **	0.54 **	0.46 **	–
Mean	23.9	29.9	48.4	19.5	2.3	12.4
SD	6.3	8.4	10.6	5.0	2.4	4.1
Alpha	0.85	0.85	0.79	0.71	0.59	0.52
Omega	0.86	0.85	0.81	0.71	0.60	0.53

** *p* < 0.001.

**Table 2:** Comparison of those who had tested positive for COVID-19 or those who lost a family member with those who had not tested positive or lost a family member.

Variable	Yes		No		*t*-Value	*p*
	Mean	SD	Mean	SD		
Tested positive?						
Perceived stress	23.66	5.83	23.45	6.43	0.25	0.805
Problem-solving confidence	29.17	7.24	29.22	8.12	−0.05	0.962
Approach-avoidance style	47.54	10.26	48.21	11.00	−0.47	0.641
Personal control	19.74	4.98	19.36	4.83	0.60	0.552
Hopelessness	2.02	2.26	2.16	2.39	−0.45	0.650
Anxiety	12.74	4.00	12.01	4.14	1.35	0.178
Lost family member?						
Perceived stress	25.27	6.10	22.93	6.23	3.34	<0.001
Problem-solving confidence	30.97	8.38	29.17	8.30	1.91	0.006
Approach-avoidance style	48.51	10.90	48.37	10.41	0.12	0.904
Personal control	19.66	4.70	19.43	5.19	0.40	0.689
Hopelessness	2.31	2.42	2.28	2.47	0.10	0.920
Anxiety	12.95	4.11	11.96	4.11	2.12	0.035

**Table 3:** Moderation analysis of the role of problem-solving appraisal in the relationship between perceived stress and psychological well-being.

Variable	Beta	SE	95% CI	*p*
Hopelessness as dependent variable				
Perceived stress	0.133	0.022	[0.090, 0.177]	<0.001
Problem-solving confidence	0.074	0.017	[0.041, 0.107]	<0.001
Approach-avoidance style	0.039	0.011	[0.016, 0.062]	<0.001
Personal control	0.124	0.028	[0.069, 0.179]	<0.001
Perceived stress X problem-solving confidence	0.005	0.002	[0.002, 0.009]	0.005
Perceived stress X approach-avoidance style	0.006	0.002	[0.003, 0.008]	<0.001
Perceived stress X personal control	0.015	0.003	[0.008, 0.021]	<0.001
Anxiety as dependent variable				
Perceived stress	0.295	0.034	[0.229, 0.362]	<0.001
Problem-solving confidence	0.141	0.026	[0.090, 0.192]	<0.001
Approach-avoidance style	0.029	0.019	[−0.007, 0.066]	0.117
Personal control	0.258	0.042	[0.175, 0.341]	<0.001
Perceived stress X problem-solving confidence	−0.005	0.003	[−0.010, 0.001]	0.121
Perceived stress X approach-avoidance style	−0.003	0.002	[−0.008, 0.002]	0.231
Perceived stress X personal control	0.000	0.005	[−0.010, 0.010]	0.951

**Table 4:** The relationship between stress and hopelessness at different levels of problem-solving appraisal.

Levels	Beta	SE	95% CI	*p*
Problem-solving confidence				
Low problem-solving confidence	0.178	0.029	[0.121, 0.235]	< 0.001
Moderate problem-solving confidence	0.133	0.022	[0.090, 0.177]	< 0.001
High problem-solving confidence	0.089	0.025	[0.040, 0.138]	< 0.001
Approach-avoidance style				
Low approach-avoidance style	0.225	0.026	[0.173, 0.276]	< 0.001
Moderate approach-avoidance style	0.166	0.020	[0.128, 0.205]	< 0.001
High approach-avoidance style	0.108	0.024	[0.061, 0.155]	< 0.001
Personal control				
Low personal control	0.225	0.028	[0.170, 0.279]	< 0.001
Moderate personal control	0.150	0.021	[0.108, 0.193]	< 0.001
High personal control	0.076	0.027	[0.023, 0.130]	0.005

## Data Availability

The data that support the findings of this study are available from the corresponding author, upon reasonable request.
